# Case report: Effective treatment of rituximab-resistant minimal change disease with obinutuzumab in an adult

**DOI:** 10.3389/fimmu.2024.1407461

**Published:** 2024-07-29

**Authors:** Qiang Wang, Lin Lin, Junhui Zhen, Bei Jiang, Guangyi Liu

**Affiliations:** ^1^ Department of Nephrology, Qilu Hospital (Qingdao), Cheeloo College of Medicine, Shandong University, Qingdao, China; ^2^ Department of Nephrology, Weifang People’s Hospital, Weifang, China; ^3^ Department of Pathology, School of Basic Medical Sciences, Shandong University, Jinan, China; ^4^ Department of Nephropathy, Qilu Hospital, Cheeloo College of Medicine, Shandong University, Jinan, China

**Keywords:** minimal change disease, obinutuzumab, refractory, rituximab-resistant, steroid-dependent

## Abstract

**Background:**

Minimal change disease (MCD) is a common cause of adult nephrotic syndrome. Most adults with MCD achieve complete remission (CR) after initial steroid therapy. However, approximately 30% of adults who respond to steroids experience frequent relapses, becoming steroid-dependent and potentially developing refractory MCD. Treating refractory MCD in adults poses a significant challenge.

**Main body:**

A 37-year-old woman presented to the nephrology department with a 6-year history of MCD. The diagnosis of MCD was confirmed via renal biopsy. She initially achieved CR with steroid treatment but experienced relapse during steroid tapering. Subsequent CR was achieved with a regimen of steroids and tacrolimus although multiple relapses occurred. Rituximab led to another CR, but its maintenance lasted only 6 months. The response to subsequent rituximab treatments was unsatisfactory. Ultimately, obinutuzumab was selected, resulting in the induction and maintenance of CR for 12 months.

**Conclusions:**

This case demonstrates the successful treatment of frequently relapsed, steroid-dependent, and rituximab-resistant MCD with obinutuzumab. Obinutuzumab is a promising therapeutic option for rituximab-resistant MCD.

## Introduction

Minimal change disease (MCD) accounts for 70–90% of nephrotic syndrome (NS) cases in children and 10–15% of idiopathic NS cases in adults. Relapse is very common in MCD, with 50% of children and 28% of adults experiencing frequent relapses. These patients are typically dependent on steroids for treatment, making their management challenging (1, 2). Cyclophosphamide, mycophenolate mofetil, rituximab, and calcineurin inhibitor are recommended for frequently relapsed (FR) and steroid-dependent (SD) patients (3). However, there are still cases that are refractory or resistant to rituximab. Obinutuzumab, a humanized type II anti-CD20 monoclonal antibody, has shown superior B cell depletion effects compared with rituximab and has been used in the treatment of chronic lymphocytic leukemia (4). It has also shown promise in refractory membranous nephropathy, rituximab-resistant mixed cryoglobulinemia, and steroid-dependent NS in children. The potential of rituximab in treating adult rituximab-resistant MCD has not been previously reported (4–6). Herein, we present a case of an adult with frequently relapsing, steroid-dependent, and rituximab-resistant MCD who achieved complete remission (CR) after obinutuzumab induction.

## Case report

**Figure 1:**
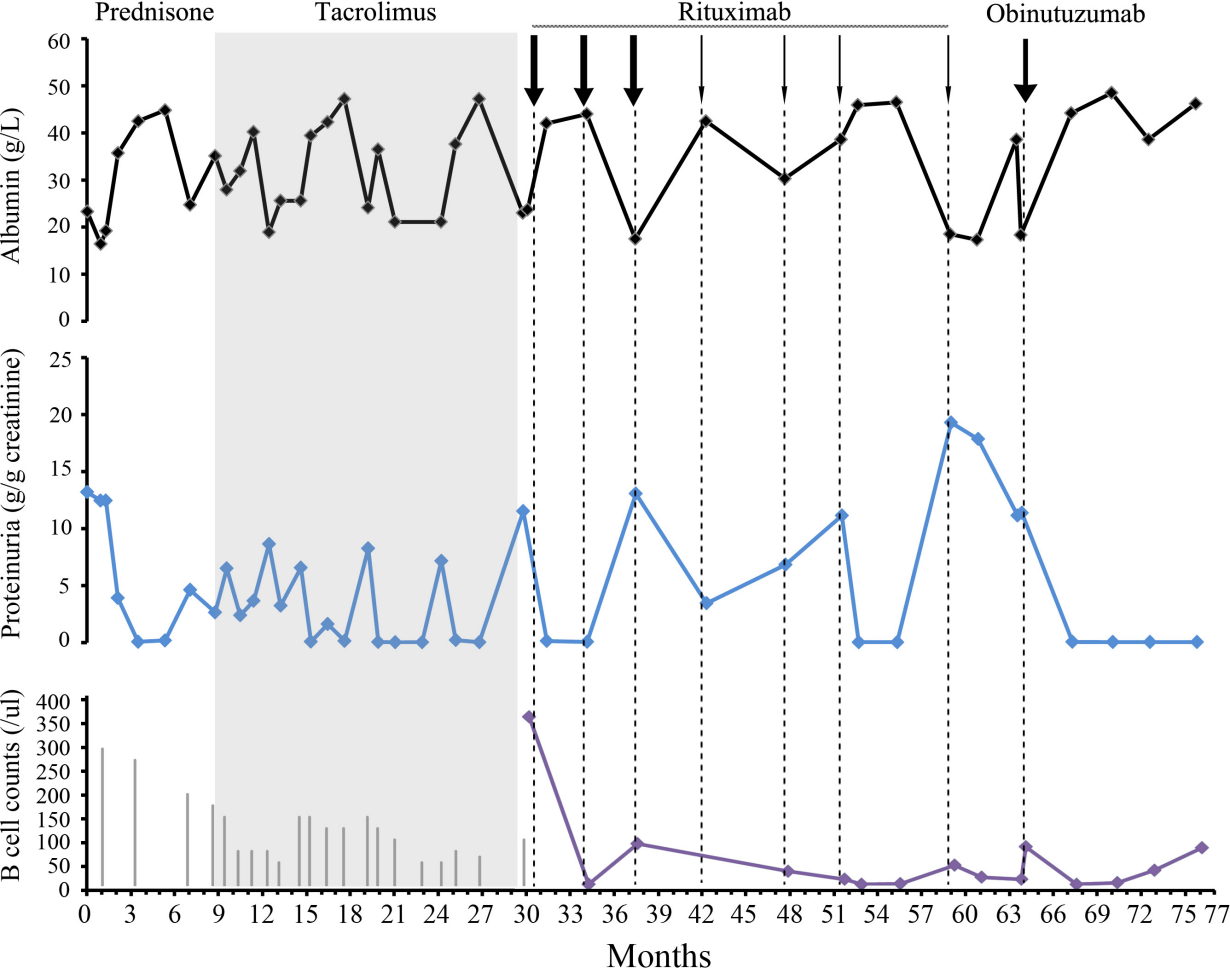
Clinical assessment and approach to treatment. The serum albumin level is indicated by the black curve. Proteinuria is evaluated using urine TPCR (blue curve). B cell counts are shown as a purple curve. The length of the gray vertical lines in the third panel corresponded to the prednisone dose. The length of the lines corresponded to the prednisone dose. The initial dose was 60 mg. The thick and thin arrows for rituximab indicate doses of 1 and 0.5 g, respectively.

A 37-year-old woman with a 6-year history of nephrotic syndrome presented to the nephrology department. She had experienced 6 relapses. The diagnosis of nephrotic syndrome was made 6 years ago after the patient developed swelling of both the eyelids and lower extremities. Physical examination revealed normotension and edema of the eyelids and lower extremities. The lung fields were normal. Initial laboratory tests showed a serum albumin level of 23.3 g/L and a urine total protein-to-creatinine ratio (TPCR) of 13.2 g/g (**Figure 1**). Urinalysis revealed no active urine sediments. The serum cholesterol level was 12.82 mmol/L. Serum urea, creatinine, and electrolytes were within normal limits. Serum phospholipase A2 receptor (PLA2R) antibody was normal. Chest X-ray and renal ultrasound were normal. She had no history of autoimmune diseases, infectious diseases, or tumors and no history of nephrotoxic medication use. No family history of renal disease was reported.

**Figure 2:**
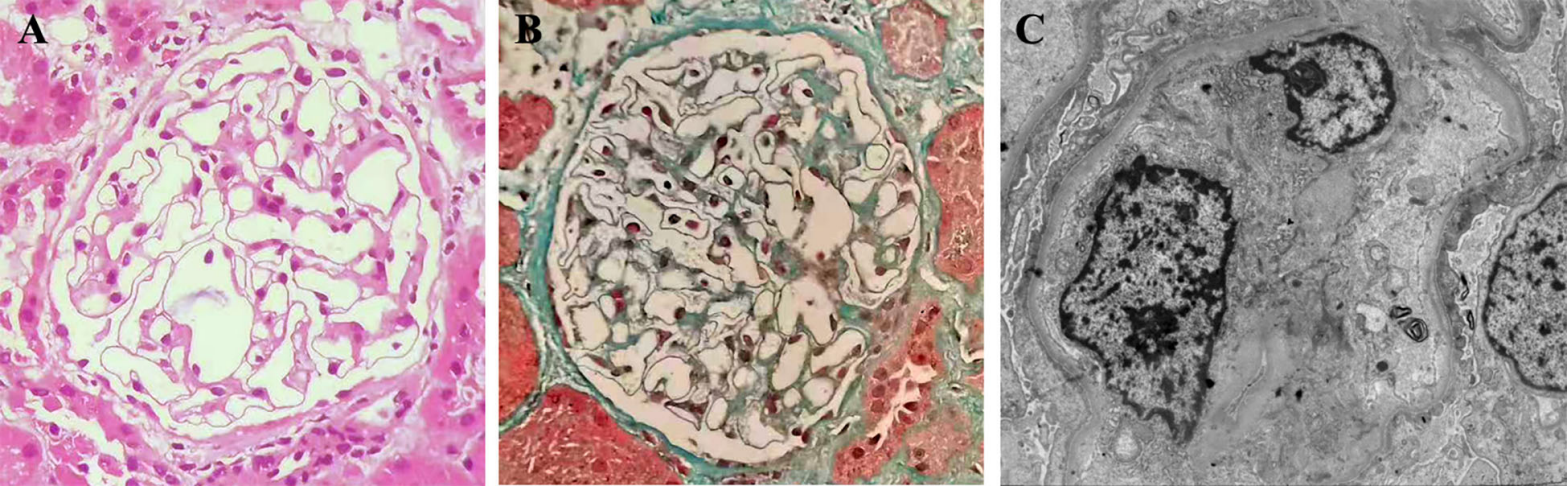
Kidney biopsy findings indicate glomerular minimal change. One of the 25 glomeruli showed signs of global sclerosis on light microscopy. The remaining glomeruli appeared normal in terms of cellularity, capillary wall, mesangial matrix, and cells [**(A)**, hematoxylin and eosin]. Masson staining did not reveal any fuchsinophilic deposits, and the structures of the arterioles appeared normal [**(B)**, masson, original magnification×200]. Immunofluorescence staining yielded negative results. Electron microscopy further revealed effacement of podocyte foot processes [**(C)**, original magnification×4000].

A renal biopsy was performed to confirm the presence of minimal change disease, as shown in **Figure 2**. Due to respiratory tract infection, she decided to postpone glucocorticoid administration. However, starting from month 2, a full dose of prednisone (60 mg) was administered. After an 8-week course of treatment, CR was achieved. Unfortunately, a relapse occurred during the 7th month while the prednisone dosage was gradually reduced. Thus, tacrolimus was initiated during the 9th month to address persistent proteinuria. The trough blood levels of tacrolimus were maintained between 5 and 8 ng/ml. Despite this intervention, three additional relapses occurred during the tapering of both prednisone and tacrolimus. Consequently, the condition was classified as steroid-dependent nephrotic syndrome (NS).

After experiencing a relapse in the 30th month, a treatment plan involving rituximab was initiated. The initial dose was 1g, followed by a second dose of 1g at 34 months. This resulted in achieving CR, which was successfully maintained for 6 months. However, a relapse occurred during the reconstitution of B cells at month 37. To address this, rituximab was re-administered at a total dosage of 2.5g from the 37th to the 51st month. This resulted in another CR accompanied by depletion of B cells, which was maintained for another 6 months until relapse occurred on the 59th month.

During physical examination, the patient exhibited normal blood pressure and lower extremity edema. Laboratory tests revealed an albumin level of 18.3 g/L and a TPCR of 18 g/g. Other serological and immune tests yielded negative results. A dose of 0.5g of rituximab was administered, but proteinuria persisted. On the 64th month, TPCR was measured at 11.38 g/g, and B cells were detected at a count of 75/ul. Despite persistent symptoms, the patient declined repeat renal biopsy and genetic testing. The condition was then categorized as refractory and rituximab-resistant MCD. Due to the patient’s desire for fertility, cyclophosphamide treatment was refused.

Subsequently, obinutuzumab was selected as an alternative treatment. A single 1g dose was administered, and the infusion process was uneventful. This led to the achievement of another CR. B cell reconstitution occurred in the 72nd month. As of the 76th month of follow-up, the patient has remained on CR.

## Discussion

This case report presents the first documented use of obinutuzumab in an adult patient with rituximab-resistant MCD, highlighting its effectiveness as a treatment option. According to the 2021 KDIGO guidelines, rituximab is recommended for frequently relapsing or steroid-dependent MCD. The precise mechanism by which anti-CD20 antibodies treat MCD is not yet fully understood. Initially, MCD was believed to be mediated by T cells, and anti-CD20 monoclonal antibodies were used to target B cell-mediated diseases. However, the efficacy of rituximab in MCD and additional research into its mechanism of action have challenged this perception.

It is now recognized that systemic immunological dysregulation underlies the biological changes seen in MCD (7–9). Depletion of B cells through rituximab disrupts a cycle in which autoreactive effector B cells and T cells mutually enhance their activity, leading to inflammation and tissue damage (10). The number of rituximab doses, the dosage administered, and the use of concurrent immunosuppressive agents can all impact the clinical efficacy in patients with MCD (11). Moreover, the development of antibodies against rituximab also contributes to the rituximab-resistant cases (12). A study has shown that up to 30% of children with SD NS who receive a single dose of rituximab may develop anti-rituximab antibodies (13).

Obinutuzumab, a third-generation humanized de-fucosylated IgG1 type II monoclonal antibody targeting CD20, has shown promising efficacy under various conditions. Compared with rituximab, it exhibits superior B cell depletion effects and is commonly used to treat chronic lymphocytic leukemia (4). The enhanced ability to deplete B cells can translate into improved clinical outcomes, as demonstrated in the NOBILITY study focusing on lupus nephritis (LN) (14).

Additionally, compared with rituximab, the distinct epitopes of obinutuzumab offer potential benefits for patients who are resistant to rituximab. Recent clinical outcomes reported by Dossier et al. highlight the use of obinutuzumab in children with steroid-resistant or steroid-dependent MCD (5). In this study, switching patients who were either resistant to rituximab or who experienced relapse after undergoing rituximab treatment to obinutuzumab resulted in successful B-cell depletion.

In this particular case, the patient experienced frequent relapses despite the use of multiple immunosuppressive agents. The introduction of rituximab after the fourth relapse did not effectively prevent future relapses. Each relapse coincided with the reconstitution of B cells, and the duration of remission progressively decreased. A meta-analysis revealed that in European studies, most relapses occurred after 6 months of rituximab treatment. However, in Asian studies in which a 500 mg of rituximab was administered every 6 months, relapses often occurred within the first 6 months. These studies identified an association between CD20 B cell recovery and relapse (12). Guitard et al. reported that CD19+ B cells reappeared before relapse at a median time of 2 months (15).

In this case, rituximab use resulted in complete depletion of B cells, suggesting the potential development of anti-rituximab antibodies. However, the absence of antibody testing prevented confirmation of this hypothesis. It is worth noting that many patients who respond to rituximab often experience relapses during the period of B-cell reconstitution. This may be attributed to the presence of resistant memory B cells encountering antigens again or the reactivation of newly generated B cells that disrupt immune tolerance (16–18). Colucci et al. found that the level of memory B cells in circulation was associated with earlier memory B-cell recovery and relapse in pediatric refractory nephrotic syndrome (19). Similarly, Bharati et al. found that the level of naïve B cells (CD19 + 27-) was lower, while the level of memory B cells (CD19 + 27+) was higher in adults with steroid-resistant MCD and focal segmental glomerulosclerosis (FSGS) (20).

Following the sixth relapse, treatment with obinutuzumab was initiated, which successfully maintained remission for 12 months. These findings suggest that obinutuzumab is effective for treating patients with rituximab-resistant MCD given its ability to maintain a longer remission. These findings could be attributed to its humanized nature and non-identical antigenic epitopes compared with rituximab (21).

One limitation of this case is that the presence of anti-rituximab antibodies was not tested, which prevented us from confirming the exact cause of the relapses. However, based on the timing of the relapses, the development of resistance antibodies may play a role.

In conclusion, obinutuzumab emerges as a promising treatment option for patients with rituximab-resistant MCD. Its efficacy in maintaining remission for a longer duration suggests its potential as a valuable therapeutic approach for this challenging condition.

## Data availability statement

The raw data supporting the conclusions of this article will be made available by the authors, without undue reservation.

## Ethics statement

The studies involving humans were approved by Ethics Committee of Qilu Hospital, Shandong University. The studies were conducted in accordance with the local legislation and institutional requirements. The participants provided their written informed consent to participate in this study. Written informed consent was obtained from the individual(s) for the publication of any potentially identifiable images or data included in this article.

## Author contributions

QW: Writing – original draft, Methodology, Data curation. LL: Writing – original draft, Data curation. JZ: Writing – review & editing, Methodology, Investigation. BJ: Writing – review & editing, Validation. GL: Writing – review & editing, Validation, Supervision.
